# Essential newborn care utilization and associated factors in Ethiopia: a systematic review and meta-analysis

**DOI:** 10.1186/s12884-020-2804-7

**Published:** 2020-02-24

**Authors:** Yoseph Alamneh, Fentahun Adane, Tadesse Yirga, Melaku Desta

**Affiliations:** 1 Department of Biomedical Sciences, School of Medicine, Debre Markos University, P.O. Box 269, Debre Markos, Ethiopia; 2 Department of Pediatrics and Child Health Nursing, College of Health Science, Debre Markos University, P.O. Box 269, Debre Markos, Ethiopia; 3 Department of Midwifery, College of Health Science, Debre Markos University, P.O. Box 269, Debre Markos, Ethiopia

**Keywords:** Ethiopia, Essential newborn care utilization, Ante/post-natal care

## Abstract

**Background:**

Globally, newborn death accounted for 46% of under-five deaths and more than 80% of newborn deaths are the result of preventable and treatable conditions. Findings on the prevalence and associated factors of essential newborn care utilization are highly variable and inconsistent across Ethiopia. Therefore, this systematic review and meta-analysis aimed to estimate the pooled prevalence of essential newborn care utilization and associated factors in Ethiopia.

**Methods:**

The international databases accessed included MEDLINE/PubMed, EMBASE, Web of Sciences, Scopus, and Grey literature databases, Google Scholar, Science Direct and Cochrane library were scientifically explored. We considered all primary studies reporting the prevalence of essential newborn care utilization and associated factors in Ethiopia. We retrieved all necessary data by using a standardized data extraction format spreadsheet. STATA 14 statistical software was used to analyze the data and Cochrane Q test statistics and I^2^ test was used to assess the heterogeneity between the studies. There significant heterogeneity between the studies so a random effect model was employed.

**Results:**

The pooled estimate of essential newborn care utilization from 11 studies in Ethiopia was 48.77% (95% CI: 27.89, 69.65). Residence [OR = 2.50 (95% CI: 1.64, 3.88)], Postnatal care [OR = 5.53, 95% CI = (3.02, 10.13], counseling during pregnancy and delivery [OR = 4.39, 95% CI = (2.99, 6.45], antenatal care follows up (OR = 6.84; 95% CI: 1.15, 4.70) and maternal educational status [OR = 1.63 (95% CI: 1.12, 2.37)] were identified as associated factors of essential newborn care utilization.

**Conclusion:**

Based on the current study essential newborn care utilization in Ethiopia was significantly low in comparison with the current global recommendation on essential newborn care utilization. Place of residence, Postnatal care, counseling during pregnancy and delivery, antenatal care follow up, and maternal educational status were associated risk factors. Therefore, on the basis of the results, it is suggested that special attention should be given to attempts to ensure that education should focus on women during ante and postnatal follow-up, counseling during pregnancy and delivery, as well as rural and illiterate mothers. Finally, appropriate newborn services at health facilities and raising mother’s level of awareness about newborn care practices are imperative in addressing the gaps in essential newborn care utilization in Ethiopia.

## Background

Globally, neonatal death accounts for about 44% of under-five mortality and Sub-Saharan Africa (SSA) has the highest rates of neonatal mortality [1]. Others report nearly 4 million newborn deaths occurred worldwide every year [2, 3]. It is estimated that 7.7 million children under the age of five die globally in a year, of whom about 3.1 million die in the neonatal period and 99% die in low- and middle-income countries [4–6]. Newborn death rates are also decreasing globally, but low and middle-income countries are experiencing much slower rate declines as compare to developed regions [5]. Although the Neonatal period contributes a greater proportion of the death rate of under-five(U5M), it is this component of under-five mortality which has shown slower reduction [7]. About 120,000 newborns die each year in Ethiopia during the neonatal period which represents 42% of all under-five mortality [8].

The use of essential newborn care according to the World Health Organization (WHO) can be described as a strategic approach to improve the health of newborns through interventions rendered prior to conception, during the time of pregnancy, during delivery and soon after birth and during the postnatal period [9]. Consequently, the WHO recommended Essential Newborn Care (ENBC) practices to reduce the risk of major causes of neonatal deaths in both community and facility delivery [10]. According to WHO recommendation, ENBC procedures include drying and wrapping the newborn immediately after birth, encouraging skin-to-skin touch, dry cord care prompt breastfeeding and delayed bathing [11]. Many studies identified various risk factors that influence the essential newborn care services utilization such as antenatal care (ANC) visits, place of delivery, mother’s level of education, counseling about ENBC, postnatal care (PNC) visits, residence, household wealth index and partner’s educational level [12–14].

Contradictory studies have been conducted in Ethiopia to determine the use of appropriate newborn care and related factors. The burden is still higher and there is plenty of uncertainty and inconsistency across regions related to the use of appropriate newborn care and its associated factors. It is therefore important to determine the pooled prevalence of ENBC and its related factors at national level and to provide a pooled estimate. The findings of this study will be used to inform, plan, implement, and evaluate relevant health promotion policies and strategies by policymakers and program planners working in the area. The study will also provide basic information for future researchers.

## Methods

### Searching strategies

The aim of this study was to determine the combined prevalence and related factors of the ENBC utilization in Ethiopia. We checked databases in this analysis without restricting on the date of publishing and designing the report. The Recommended Reporting Items of the Systematic Reviews and Meta-Analysis Protocol (PRISMA-P) guidelines have been used to validate scientific accuracy [15]. The international databases included MEDLINE/PubMed, EMBASE, Web of Sciences, Scopus, and Grey literature databases, Google Scholar, Science Direct and Cochrane Library were scientifically explored.

Additionally, to obtain additional articles, we checked reference lists of established studies. Unpublished studies were retrieved from the official websites of international and local organizations and universities. Keywords, medical subject headings (MeSH) terms were used to conduct the search. We used the search terms independently and/or in combination using “OR”, “AND” or “NOT”. Keywords/search terms were “Prevalence” OR “Epidemiology” AND “essential” AND/OR “essential newborn care” OR “essential newborn” AND/OR “essential newborn care” AND/OR “essential newborn care” AND “utilization” AND/OR “services” AND “factors” AND/OR “associated factors” AND/OR “risk factors” AND/OR “determinants” AND/OR “predictors” AND” Ethiopia”. All articles were conducted from August 30, 2019, to September 30, 2019, and all accessible studies up to September 30, 2019, were incorporated in our meta-analysis and systematic review.

### Identification and selection of studies

This meta-analysis and systematic review included studies in both institutional and community-based studies that reported the use of ENBC and related factors in Ethiopia. This review included all articles published in peer-reviewed journals, that were written in English. We excluded any primary studies considered, inaccessible for full-text article after contacting the primary author twice via email, and in case of our outcome of interest did not respond. All studies that reported the prevalence of ENBC services utilization and its determinants in Ethiopia were included.

### Data extraction and synthesis

Data were retrieved by two independent reviewers using a standardized data extraction spreadsheet format. The data abstraction format includes author, study year, region of study setting (region and rural or urban), study design, sample size, prevalence, and associated factors. Any disagreements during the extraction process were resolved by consensus between the reviewers. In instances of incomplete data, we excluded the study after two attempts were made to contact the corresponding author by email. Also, the two authors performed the quality assessment of studies independently. Any discrepancy was resolved by discussion and agreement.

### Quality assessment of the studies and risk of bias assessment

To assess the quality of each study, we applied the Newcastle-Ottawa quality assessment tool scale adopted for cross-sectional studies [16]. The modified the Newcastle – Ottawa scales consists of three sections. The first section tool is rated up to five stars for methodological evaluation. The second section tool is ranked up to three stars for comparability assessment. The third section tool is evaluated up to two points that deal with the statistical analysis and the outcome of each study. The original study was assessed by two reviewers independently and any disagreement between the reviewers was resolved by taking the mean score of the two reviewers. Finally, the original studies with the scale of ≥6 out of 10 were considered as high quality after reviewing different literature.

### Data synthesis and statistical analysis

For further analysis, we imported the data into STATA Version 14.0 statistical software after extracting the data using Microsoft^MT^ Excel format. Using the binomial distribution formula, Standard error was calculated for each study. We identify the heterogeneity between the studies using Cochrane’s Q statistics (Chi-square), inverse variance (I^2^), and *p*-values [17]. The statistical output showed that there was significant heterogeneity among the studies (I^2^ = 99.8%, *p* = 0.000) so a random-effects meta-analysis model was used to estimate the pooled prevalence and associated factors of ENBC utilization in Ethiopia. A forest plot to detect the presence of heterogeneity. Furthermore, subgroup analysis and meta-regression were used to identify the possible source of heterogeneity. The evidence of publication bias was checked using funnel plot symmetry. Besides, the statistical significance of publication bias was assessed using both Egger’s and Beggar’s test, subsequently, a trim-and-fill analysis was performed, with the *p*-value, less than 5% used to declare the presence of publication bias [18, 19].

## Results

### Study selection and data extraction

**Fig. 1:**
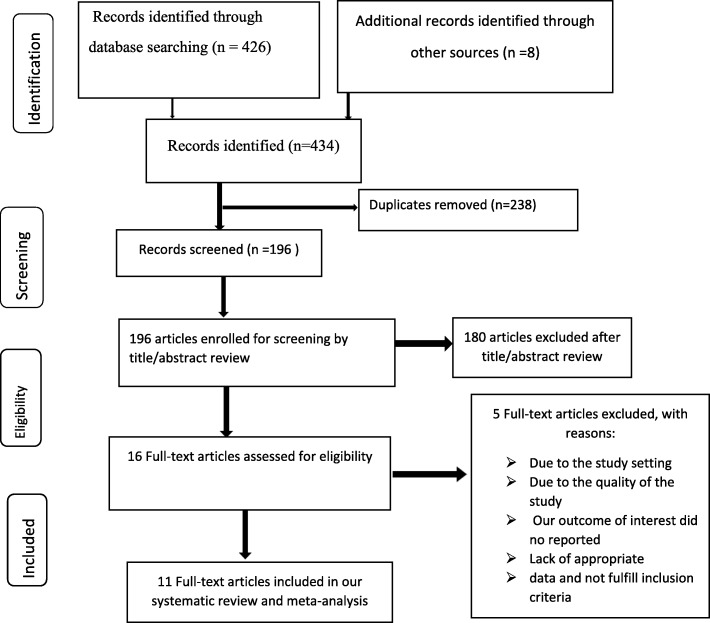
PRISMA Flow Diagram of Included Studies to Estimate the Pooled Prevalence and Associated Factors of Essential Newborn Care Utilization in Ethiopia, 2019

A total of 434 studies were identified using electronic searches (through Database searching (*n* = 426)) and other sources (*n* = 8)) that was conducted from August 30, 2019, up to September 30, 2019. Of these, 238 studies were excluded due to duplication. From the remaining 196 studies, after reviewing the title and abstract 180 studies were excluded as they were irrelevant for this systematic review and meta-analysis. The remaining 16 full-text articles were assessed for eligibility criteria based on the pre-defined criteria. Among these five articles were further excluded due to they are not inline to the preset criteria, three studies from Ethiopia [20–22], one study from Nepal [23] and one study from Himalayas [24]. Finally, 11 studies fulfilled the eligibility criteria and included in this systematic review and meta-analysis **(**Fig. 1**)**.

### Characteristics of the studies

A total of 5416 Study participants were included in his systematic review with a range of 296 in Tigray [25] to 845 in Amhara [26] in individual studies carried out between 2015 to 2019.

### Utilization of essential newborn Care Services in Ethiopia

**Fig. 2:**
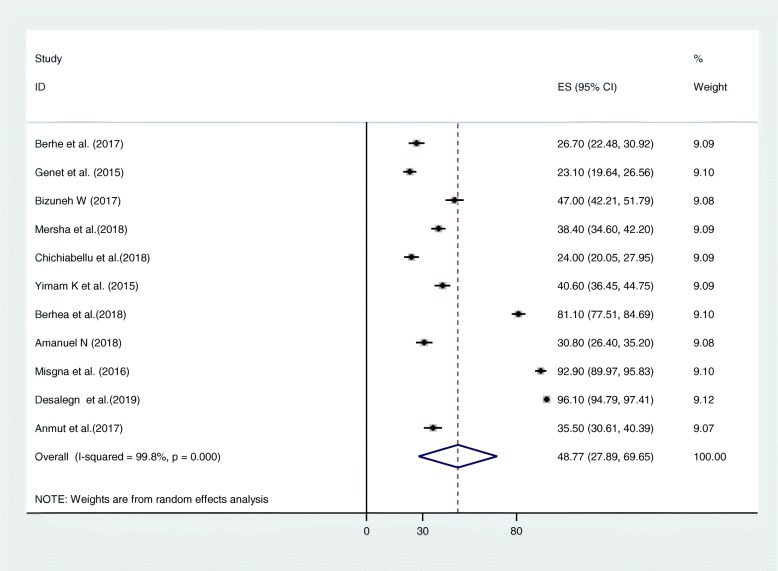
Forest Plot for The Prevalence of Essential Newborn Services Utilization in Ethiopia, 2019

The utilization of ENBC services from included studies ranged between 23.1 and 96.1% (Table 1). As indicated in the forest plot, the pooled estimate for utilization of ENBC services from 11 studies in Ethiopia was 48.77% (95% CI: 27.89, 69.65) **(**Fig. 2). We identified a high and significant heterogeneity between studies (I^2^ = 99.8%%; *p*-value = 0.000), indicating great variability in utilization across studies so a random effect analysis model was used to estimate the pooled prevalence of the utilization of essential newborn care service in Ethiopia (Fig. 2**).** We performed a subgroup analysis based on study area and study setting to identify the source of heterogeneity (Table 2). Beyond subgroup analysis, meta-regression for the included studies was conducted to identify factors for heterogeneity. However, there was no statistical significance from the meta-regression (Table 3).

**Table 1:** Characteristics of 11 Studies Reporting the Essential Newborn Care Utilization and Its Associated Factors in Ethiopia, 2019

SN	Author and Year of Publication	Study Area	Place setting	Study Design	Sample size	Prevalence (95% CI)	Quality Score
1	Berhe et al., 2017 [2]	Tigray	Institution-based	Cross-sectional	423	26.70 (22.48, 30.92)	8
2	Genet et al., 2015 [19]	Amhara	Community Based	Cross-sectional	570	23.10 (19.64, 26.56)	9
3	Bizuneh W., 2017 [20]	Oromia	Institution Based	Cross-sectional	417	47.00 (42.21, 51.79)	7
4	Marsha et al., 2018 [21]	SNNPR	Community Based	Cross-sectional	630	38.40 (34.60, 42.20)	8
5	Chichiabellu et al., 2018 [13]	SNNPR	Community Based	Cross-sectional	450	24.00 (20.05, 27.95)	9
6	Yimam K et al., 2015 [22]	Benishangul Gumz	Community Based	Cross-sectional	539	40.60 (36.45, 44.75)	7
7	Berhea et al., 2018 [23]	Tigray	Community Based	Cross-sectional	456	81.10 (77.51, 84.69)	9
8	Amanuel N. 2018 [24]	SNNPR	Community Based	Cross-sectional	422	30.80 (26.40, 35.21)	9
9	Misgna et al., 2016 [25]	Tigray	Community Based	Cross-sectional	296	92.90 (89.97, 95.83)	8
10	Desalegn et al., 2019 [26]	Amhara	Community Based	Cross-sectional	845	96.10 (94.80, 97.41)	7
11	Anmut W, et al., 2017 [27]	SNNPR	Community Based	Cross-sectional	368	35.50 (30.61, 40.39)	9

**Table 2:** Subgroup Analysis Which Describes the Pooled Prevalence of Essential Newborn Care Utilization in Ethiopia, 2019

Subgroup		Included Studies	Prevalence (95% CI)	Heterogeneity statistics	*P* value	I^2^ (%)	Tau-squared
Region	Tigray	3	66.93 (29.82, 104.03)	658.71	0.000	99.7%	1.1e+ 03
	Amhara	2	59.62 (−11.92, 131.16)	1496.90	0.000	99.9%	2.7e+ 03
	SNNPR	4	32.15 (25.59, 38.72)	28.95	0.000	89.6%	40.1705
Study setting	Institution-based	2	36.82 (16.92, 56.71)	38.88	0.000	97.4%	200.7448
	community Based	9	51.41 (28.25, 74.57)	3919.17	0.000	99.8%	1.3e+ 03

**Table 3:** Meta-Regression for The Included Studies to Identify the Source of Heterogeneity for The Essential Utilization of Newborn Care Service in Ethiopia, 2019

Variables	Characteristics	Coefficient	*P*-value
Year	Publication year	5.779168	0.420
Sample size		.0388121	0.536
Region	Tigray	26.33038	0.472
	Amhara	19.05547	0.619
	Oromia	6.400002	0.884
	SNNP	−8.426123	0.808
	Benishangul Gumuz	Reference	Reference
Study setting	Institution-based	−14.58638	0.530
	Community-based	Reference	Reference

### Publication bias

We observed publication bias using both Begg’s and Egger’s tests [18, 19] with these tests yielding statistical evidence of publication bias at a *p*-value less than 0.05 and the funnel plot was asymmetry. In considering publication bias trim and fill meta-analysis was done [27]. However, based on this analysis, the prevalence of ENBC utilization was 48.77 and no significant change was seen as compared with the main meta-analysis.

### Sensitivity and subgroup analysis

Due to considerable heterogeneity in this review, Subgroup analysis was done by setting of studies and regions. Based on Subgroup analysis report the pooled prevalence of ENBC utilization was higher in Tigray region (66.93%) followed by Amhara region (59.62%). Subgroup analysis was also carried out based on the study setting, a prevalence rate of 51.41 and 36.82% which revealed from community based and institution-based respectively **(**Table 2**).** A sensitivity analysis was done to identify outlier studies. According to the analysis, no influential studies were detected so all of the studies were included in the final analysis.

### Associated factors of newborn care service utilization

**Fig. 3:**
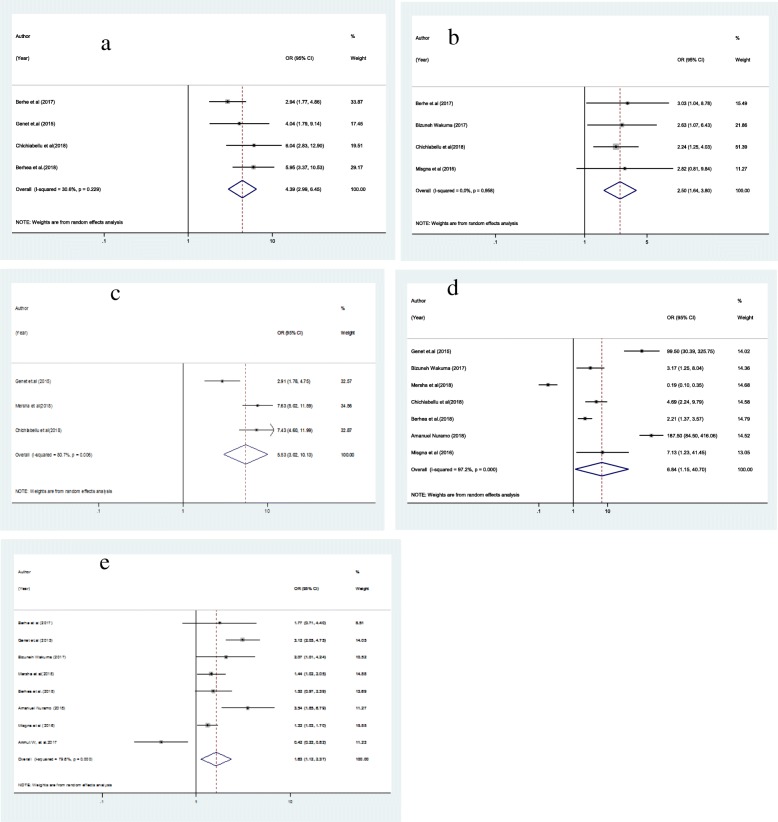
Forest Plot Showing Pooled Odds Ratio of the Associated Factors for Essential Newborn Care Utilization. **a** Counseling About ENC Practice During Delivery, **b** Urban Residence, **c** Postnatal Care, **d** Antenatal Care, **e** Maternal Educational Status

A total of 11 studies were included for analysis of an associated factors of ENBC utilization. We identified five main associated factors with the pooled odds ratio ranging from 1.63 to 6.84. These associated factors were place of residence, immediate PNC, counseling during delivery, ANC follows up and educational status of mothers. The analysis of 11 studies showed that counseling about ENBC practices was showed statistically significant association with ENBC practice of mothers as compared with those mothers who had not receive counseling about ENBC practices during ANC, and PNC follow up [OR = 4.39, 95% CI: (2.99, 6.45] (Fig. 3a). Similarly, this study showed that living in urban centers was strongly associated with ENBC utilization [OR = 2.50 (95% CI: 1.64, 3.88)] (Fig. 3b**).** Furthermore, the PNC visit yielded a statistically significant association with ENBC practice of mothers when compared with those who had no immediate PNC visits after delivery [OR = 5.53, 95% CI: (3.02, 10.13] (Fig. 3c). Moreover, utilization of newborn care services showed statistically significant association with ENBC among mothers who had two or more ANC visits compared to mothers with no ANC visits (OR = 6.84; 95% CI: 1.15, 4.70) (Fig. 3d). Mothers who had formal educational status were 1.63 times more likely to practice ENBC as compared to those who had no formal education [OR = 1.63 (95% CI: 1.12, 2.37)] (Fig. 3e).

## Discussion

This research is important for understanding current newborn care practices and factors that affect them in order to intervene to increase the satisfaction and use of maternal and neonatal health services by individuals, families and communities, and for policymakers to establish criteria for improving the quality of maternal and newborn care in health care facilities. Despite the World Health Organization’s recommendation for the first 6 months of life on exclusive breastfeeding (EBF), the rate remains low in both developed and developing countries, including Ethiopia [28, 29]. This may be because women need effective support for breastfeeding but many health care workers lack the necessary knowledge, attitudes and skills. It is therefore important to train health care professionals on essential neonatal care practice and to encourage breastfeeding with a positive short- and long-term impact on the health of both children and women. Implementation of evidence-based neonatal care would increase the use of established education programs, adaptable to local environments and deliverable using a train-the-trainer model.

In this meta-analysis, we extensively reviewed studies analyzing the use of ENBC services within Ethiopia and related variables. This systematic review and meta-analysis was aimed at estimating the pooled prevalence of the use of ENBC in Ethiopia and its predictors. The combined estimate from 11 studies in Ethiopia for the use of ENBC services was 48.768%.This finding was lower than the studies done in Nepal 70.7% [30], and India 66.70% [31], quite possibly due to socio-economic differences between the study areas, socio-cultural variation, the study period and setting, sample size and/or target population. Other possible explanations may be due to maternal health services coverage varying in different countries based on increased awareness and information about ENBC utilization.

Our study findings revealed that counseling about ENBC practices among mothers during and after pregnancy were statistically significant association with ENBC practice of mothers as compared with those not seeking advice on ENBC practices during ANC and PNC. This can be explained by the fact that mothers who received training on essential newborn care during ANC, delivery and PNC periods could better understand the importance of the practice of ENBC [32]. There may be other possible reasons community health workers (HEWs) could discuss and educate about ENBC during monthly meeting [33]. Thus, to support the utilization of health facilities for prevent and treat neonatal mortality and morbidity, prompt postnatal care (PNC) for the mother and the child is important.

In our study, those mothers whose educational status is primary and above are more likely to practice ENBC as compared with those mothers who are not able to read and write. This finding aligns with the research done in Nepal in which educational status was one of the predictors of essential newborn care practice [30]. This might be related to the fact that educated mothers may have a better understanding of ENBC practices. Additionally, maternal knowledge of essential newborn care must start with an effective educational plan before the baby’s birth [34] and identified that higher levels of parental education have a significant impact on the level of knowledge about newborn care. Again these findings were similar to those reported in India and Nepal [35, 36]. Urban residence of mothers have been strongly associated with the use of appropriate newborn care with a 2.50 likelihood compared to rural residents, yielding a similar finding to a study conducted in Sri Lanka [37]. It is possible that health service accessibility and good secondary knowledge to enhance urban mother’s educational status compared to women in rural areas, and urban residents get information more readily and frequently about ENBC utilization than their rural counterparts [12]. The current review of utilization of newborn care services showed a statistically significant association with ENBC among mothers who had two or more ANC visits compared to mothers making no visits. This finding was supported by other study conducted in Northern Ghana [13]. The possible justification could be that mothers who attended ANC have the chance of getting information about the components and the importance of newborn care practice from health professionals. Additionally, ANC is positively associated with clean cord care and thermal care practices [35]. Furthermore, immediate PNC visit had a statistically significant association with ENBC practices of women in this study. This could relate to health workers advising on the ENBC care during immediate PNC visits which equates to proper, prompt, and appropriate advice in a timely and meaningful period of time. A Study done in Italy and Namibia showed that socio-demographic characteristics such as marital status have been found to be significantly associated with maternal health care service utilization and essential newborn care women who were single, divorced or widowed were less likely than married women to use maternal health care service and essential newborn care [38, 39]. Surprisingly, almost all studies conducted in Ethiopia did not consider marital status as a consideration for mothers with access to healthcare facilities and obstetric outcomes that directly affect the practice of essential newborn care, so that future researchers should explore marital status as positively related to access and use of maternal healthcare and essential newborn practice.

### Limitations

The results of this systematic review and meta-analysis had limitations: in this systematic review and meta-analysis, all articles considered were cross-sectional in nature. As a consequence, it is not possible to establish temporal relations between factors and outcome variables. Most of the research included in this review had a small sample size that could influence the final estimate. Furthermore, since this meta-analysis included accessible research recorded from a small number of institutes in Ethiopia, the various areas in the nation may be under-represented.

## Conclusion and recommendation

Based on this systematic review and meta-analysis, ENBC utilization in Ethiopia was significantly low as compared to the current global recommendation on ENBC utilization. Place of residence, Postnatal care, counseling during pregnancy and delivery, Antenatal care follow up and maternal educational status were the predictor variables. Therefore, on the basis of the results, it is suggested that special attention should be given to attempts to ensure that education should focus on women for ante and postnatal care follow-up, counseling during pregnancy and delivery, as well as rural and illiterate mothers. Utilization of essential newborn care practice can reduce newborn death by 80%. Hence, appropriate newborn services utilization and distribution of the resources should be ensured, to sustain the more vulnerable populations at health facilities and raising mother’s level of awareness about newborn care practices and more emphasis should be given by concerned bodies.

## Data Availability

The datasets analyzed during the current study are available from the corresponding author upon reasonable request.

## Abbreviations

ANC: Antenatal care
CI: Confidence interval
ENBC: Essential newborn care
OR: Odds ratio
PNC: Postnatal care
SNNP: Southern Nations, Nationalities, and Peoples
SSA: Sub-Saharan Africa
U5M: Under five mortalities
WHO: World Health Organization
